# Herpesviruses: Harmonious Pathogens but Relevant Cofactors in Other Diseases?

**DOI:** 10.3389/fcimb.2018.00177

**Published:** 2018-05-25

**Authors:** Sharvan Sehrawat, Dhaneshwar Kumar, Barry T. Rouse

**Affiliations:** ^1^Department of Biological Sciences, Indian Institute of Science Education and Research Mohali, Mohali, India; ^2^Department of Biomedical and Diagnostic Sciences, College of Veterinary Sciences, The University of Tennessee, Knoxville, Knoxville, TN, United States

**Keywords:** herpesviruses, cofactors, host–pathogen interactions, disease outcome, virome, coinfections

## Abstract

Most vertebrates are infected with one or more herpesviruses and remain so for the rest of their lives. The relationship of immunocompetent healthy host with herpesviruses may sometime be considered as harmonious. However, clinically severe diseases can occur when host immunity is compromised due to aging, during some stress response, co-infections or during neoplastic disease conditions. Discord can also occur during iatrogenic immunosuppression used for controlling graft rejection, in some primary genetic immunodeficiencies as well as when the virus infects a non-native host. In this review, we discuss such issues and their influence on host-herpesvirus interaction.

## Introduction

**Figure 1:**
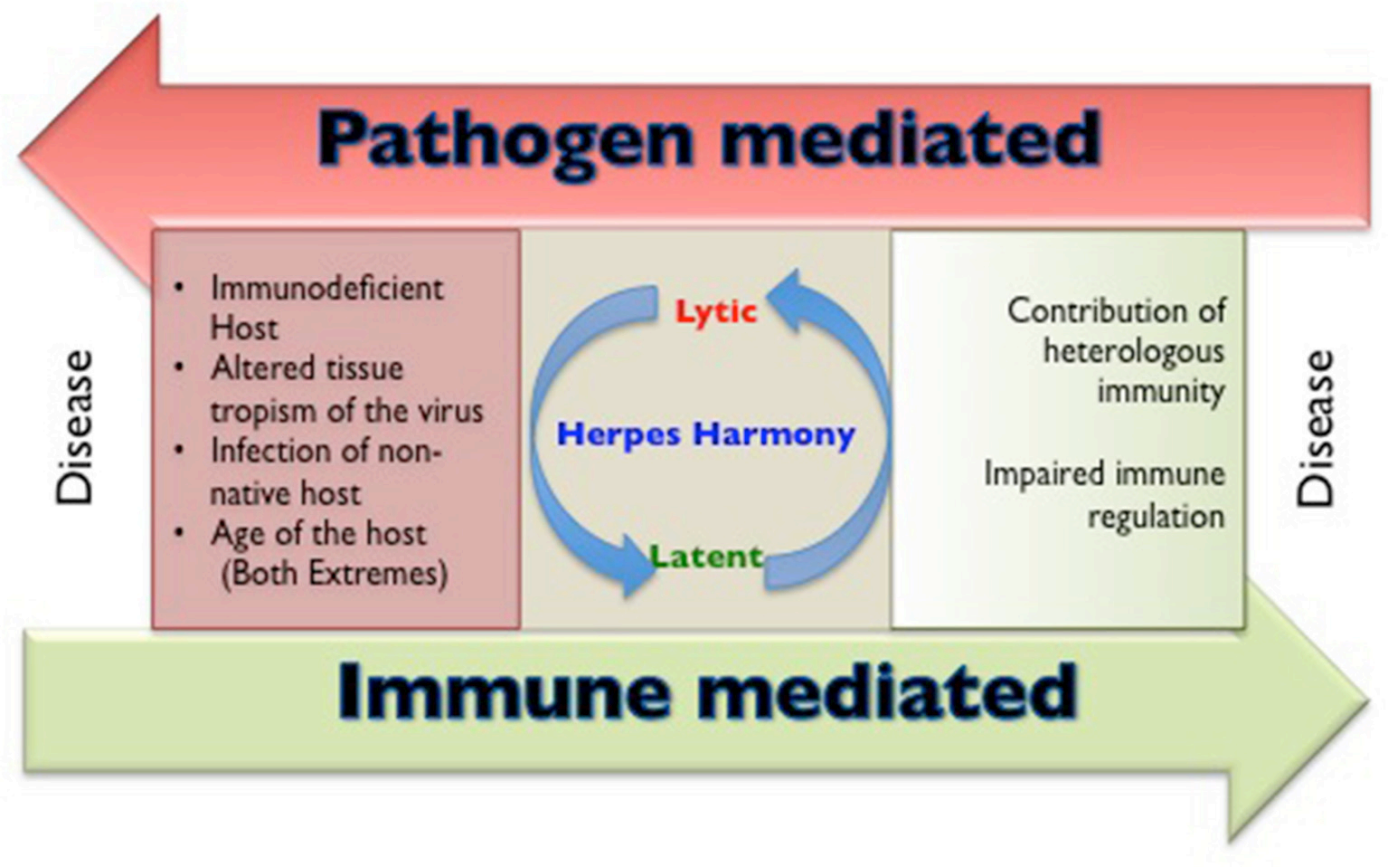
A cartoon to show the circumstances that are responsible for making and breaking herpesviruses and host harmony.

The members of *herpesviridae* family are categorized into alpha (α), beta (β), and gamma (γ) herpesviruses based on their host range, genetic organization and replication strategies (Whitley, 1996). Herpes simplex virus (HSV) 1, 2 and varicella zoster virus (VZV) are α-herpesviruses, cytomegalovirus (CMV), human herpesvirus (HHV)−6 and 7 are β-herpesviruses while Epstein Barr Virus (EBV) and human herpesvirus 8 (HHV8) are γ-herpesviruses infecting humans. All humans become infected with one or more herpesviruses during their life span (Boshoff and Weiss, 2001; Virgin et al., 2009). Characteristically, herpesviruses persist in the host for an extended duration following a primary infection, but severe disease and mortality in healthy immunocompetent individuals caused by α- and γ-herpesviruses are rare. However, CMV infection involving critical organs of nervous system, hematological and vascular system, gastrointestinal system may be accompanied by severe disease outcome in apparently healthy individuals (Rafailidis et al., 2008). The influence of any unaccounted for critical conditions remain a possibility in such cases. The general perception is that herpesviruses are innocuous pathogens, a status that can probably be attributed to their long association with mankind (Parrish et al., 2008). In some instances, the persisting herpesvirus infections might even provide some benefits to the host against other infections and clinical conditions such as malignancies (Barton et al., 2007; White et al., 2012; Furman et al., 2015; Litjens et al., 2018). Humans not infected with any of the herpesviruses however, represent a rare subset; therefore in comparison to infected individuals their ability to handle other infections is not well-established. The outcome of herpesvirus infection is severe in genetically immunodeficient, very young or aged individuals as well as when the virus gains entry to certain anatomical locations such as central nervous system or other immunoprivileged sites. In addition, when herpesviruses infect either a non-native susceptible host or those organisms that harbor other concurrent infections, severe disease may occur (Figure 1). Evidence that co-infection of HSV and human immunodeficiency virus (HIV) can result in more severe disease outcome is well established (Freeman et al., 2006; Des Jarlais et al., 2014; Looker et al., 2017). In this review, we discuss situations and underlying cellular and molecular mechanisms where the disease pattern caused by herpesviruses is changed. We focus our analysis on α-herpesviruses while briefly alluding to the other members of the herpesviridae family throughout the text.

## Dynamics of host and herpesviruses interaction

Herpesviruses are considered as highly successful pathogens. These viruses might have originated from those viruses that infected a common ancestor of mammals, birds and reptiles (McGeoch et al., 1995; Virgin et al., 2009; Virgin, 2014). The evolutionary processes led to the selection of variants with altered infectivity and tissue tropism probably to ensure survival and propagation. The sequence analyses of HSV1 and HSV2 revealed that HSV1 might have infected the ancestors of humans much earlier than HSV2 and therefore has evolved better to persist in human host while HSV2 was introduced in humans at a later time point through an intermediate host. Therefore HSV2 may not had enough time to evolve with the host (Parrish et al., 2008; Wertheim et al., 2014; Underdown et al., 2017). This temporally distinct but longer association of HSV1 with human host could possibly explain why HSV1 is less pathogenic than HSV2 (Sedarati and Stevens, 1987; Smith T. J. et al., 2002). In general, herpesviruses have better adapted for their human host as compared to many other categories of viruses and therefore can persist in the host.

Numerous properties of herpesviruses contribute to their success and these have been discussed in detail by others (Whitley, 1996; Ploegh, 1998; Kapadia et al., 2002; Orange et al., 2002). The most important characteristic is their ability to adopt two different modes of life cycle; the latency and the lytic cycles. Herpesviruses after a primary productive infection resort to latency, a transcriptional and translational suppressed state. However, the latent stage is frequently interrupted by clinically asymptomatic reactivation episodes (Stevens, 1989; Wald et al., 2002; Kelly et al., 2006; Mark et al., 2008; Nicoll et al., 2012; Roizman and Whitley, 2013; Uppal et al., 2014; Virgin, 2014). Neurotropic viruses such as HSV 1, 2 and VZV preferentially establish latency in neuronal cells but one particular region of the virus genome remains transcriptionally active and produces latency-associated transcripts (LATs) (Roizman and Whitley, 2013). LATs are well characterized for HSV 1 and 2 but not so well for VZV (Leib et al., 1989; Strelow and Leib, 1995; Depledge et al., 2017). Functional protein products are rarely detected during latency (Simmons et al., 1992; Roizman and Whitley, 2013). Moreover most neurons lack MHC molecules and hence are unlikely to stimulate the immune system even when limited viral proteins are made under some circumstances (Maehlen et al., 1989). The immune cells or other non-neuronal cells in the vicinity of infected neurons could still acquire such antigens and help trigger or maintain immune reactivity. The overall kinetics, the magnitude and the contribution of these immune induction processes are not well understood. The primary infection of neurons by α-herpesviruses, results in the lysis of a significant proportion of neurons that may cause neuritis or post herpetic neuralgia in some infected individuals (Tontodonati et al., 2012). Fewer neurons are damaged during intermittent reactivation or abortive replication events in immunocompetent host and in such situations clinical symptoms usually do not occur (Antinone and Smith, 2010; Sawtell and Thompson, 2016).

Unlike α-herpesviruses, β- and γ-herpesviruses such as CMV and EBV, establish latency in non-neuronal cells that include macrophages and B cells (Klein et al., 1989; Slobedman and Mocarski, 1999; Kelly et al., 2006; Sinclair and Sissons, 2006; Khairallah et al., 2017). Murine cytomegalovirus (MCMV) infection in mice represents one of the well-studied β-HV model system to understand the host pathogen interaction. This infection effectively induces both innate and adaptive immunity. One of the proteins (m157) encoded by MCMV serves as a ligand for receptor (Ly49H) expressed by NK cells. This interaction induces the activation and differentiation of such cells in a manner CD4^+^ and CD8^+^ T cells are signaled. Some of the responding NK cells can even form a memory pool (Sun et al., 2009). The conventional αβ- as well as a less-well characterized γδ-T cell response help control the balance of lytic and latent virus (Steffens et al., 1998; Couzi et al., 2015; Khairallah et al., 2017). The entire repertoire of γδ-T cells is not exhaustively studied but nonetheless include antigenic moieties from diverse sources such as proteins, lipids, lapidated peptides, small molecules and antigen recognition by these cells may be dependent on class I MHC molecules or other non-classical MHC like molecules such as CD1c (Chien et al., 2014). Such cells were also shown to provide protection to MCMV infected mice independently of αβ-T cells (Khairallah et al., 2015). Many viral proteins are expressed in EBV infected B cells during the non-productive cycle. Many excellent reviews and research articles can be referred to for further insights (Kelly et al., 2006; Speck and Ganem, 2010; Nicoll et al., 2012; Roizman and Whitley, 2013; Khairallah et al., 2017). We highlight some of the generally accepted key points during herpesvirus latency.

The microenvironment, cell autonomous factors as well as viral elements may all contribute to the latency establishment, maintenance and reactivation of herpesviruses. Among viral factors, LATs predominantly regulate herpes simplex virus latency. LATs can actively regulate the expression of viral lytic genes such as immediate early protein encoding genes (ICP0, ICP4) and thymidine kinase of HSV to limit reactivation (Kramer and Coen, 1995; Chen et al., 1997; Roizman and Whitley, 2013). LATs can also interfere with cellular metabolism and inhibit the caspases dependent apoptosis of infected cells (Perng et al., 2000; Henderson et al., 2002; Roizman and Whitley, 2013). Some have suggested that epigenetic modifications in HSV genome differ during latency and lytic cycles (Bloom et al., 2010). In general, the association of HSV genome with nucleosomes and modification of histones by methylation and acetylation at specific lysine residues were shown to determine the lytic and latent viral replication cycles (Knipe and Cliffe, 2008; Bloom et al., 2010). The sampled tissues invariably contain both latently infected and some neurons undergoing recent reactivation of viral genome. Therefore, the results thus obtained are difficult to interpret but the technologies that offer analyses on identifiable single cells might provide better insights.

Nerve growth factors (NGF) produced by multiple cell types could also be involved in latency (Wilcox et al., 1990). NGF signaling up-regulates Bcl2 to promote cellular proliferation and survival (Finkbeiner, 2000; Biswas and Greene, 2002). Nerve termini express NGFs and physical damage could change NGF levels and possibly precipitate viral reactivation (Wilcox et al., 1990; Wilson and Mohr, 2012). This could possibly explain why some people suffer from frequent HSV reactivation upon mechanical or infection induced injuries to the skin which is an extensively innervated organ (Hsieh et al., 1997). The herpesvirus encoded miRNAs or a modulation of host miRNAs by herpesvirus infection can influence different aspects of latency (Rezaee et al., 2006; Umbach et al., 2008; Du et al., 2015; Grey, 2015). Herpesvirus encoded miRNAs help restrict replication and favor latency (Umbach et al., 2008). Examples include miR-UL112-1 of HCMV and miR-K12-9, which target the immediate early transactivator, IE72 of HCMV and RTA of KSHV, respectively, to interfere with viral replication (Grey et al., 2007; Bellare and Ganem, 2009). This interference with viral replication ensures that the latency is maintenained. Several cellular miRNAs that are either expressed at a basal level or induced upon infection may also influence viral gene expression (Lecellier et al., 2005). An intriguing example is miR138, which is mainly expressed by neurons and targets ICP0 of HSV to block the replication cycle and thereby facilitating latency (Pan et al., 2014). There are also examples of host miRNA such as miR23a which promotes the lytic life cycle by interfering with the activity of interferon regulatory factors (IRFs) to compromise the antiviral state in infected cell (Ru et al., 2014). Some host miRNAs could interfere with cellular metabolism. Example is miR101, whose ectopic expression in latently infected cells blocked HSV1 replication while its depletion promoted viral replication. This miRNA directly interacted with one of the genes whose product is required for ATP production (Zheng et al., 2011). Therefore, the expression kinetics of different miRNAs in infected cells may help decide between a lytic cycle and latency.

Upon viral infection both effector and regulatory cells are recruited in the response. The magnitude and nature of such cells may influence the switch between the lytic cycle and latency. How these cells are maintained in infected ganglionic tissues is still not clearly understood (Lund et al., 2008; Veiga-Parga et al., 2013). Thus, during latency, the first signal (peptide-MHC) required to activate T cells may not be available to maintain the pool of viral reactive T cells. Some studies suggest that even during latency, a limited number of neurons permit viral replication to provide antigenic stimulation for T cells (Wald et al., 2002; Wald and Corey, 2007). The role of T cells in regulating herpesvirus latency is frequently studied in mice models as samples obtained from human cadavers might display pronounced viral reactivity (Ouwendijk et al., 2012). However, one of the key HSV immune modulators, ICP47, fails to block the antigen presentation machinery by interacting with mouse transporter of antigen processing and presentation (TAP) molecules unlike its human counterpart (Tomazin et al., 1998; Verweij et al., 2011). This suggests for a limited direct applicability of mice studies in humans. Extensive studies supporting a role for CD8^+^ and perhaps CD4^+^ T cells have mainly come from the Hendricks laboratory (Liu et al., 2000, 2001; Knickelbein et al., 2008). Basically, those studies implicate the role for granzyme B and interferon gamma (IFN-γ) produced by virus specific CD8^+^ T cells with the major viral glycoprotein, gB and some early replication proteins providing the peptides for T cell recognition. Both these immune mediators are responsible for inhibiting viral replication (Cantin et al., 1995; Liu et al., 2000, 2001; Knickelbein et al., 2008; Treat et al., 2017). Granzyme B cleaves viral protein ICP4 to cause abortive replication events (Knickelbein et al., 2008). In fact, an abundance of virus-specific activated CD8^+^ T cells that produce effector molecules were found in latently infected ganglionic tissues and a large majority of these produced effector molecules such as granzyme B and IFN-γ (Wilson and Mohr, 2012). Whether local microenvironment provides sufficient signals to maintain viral reactive T cells or secondary lymphoid organs are continuously feeding infected ganglionic tissues remain poorly understood. Regulatory T cells are also found in latently infected ganglionic tissues and these cells probably help control the hyper reactivity of immune cells that may kill irreparable neurons (Suvas et al., 2006). However, some studies have suggested that the microenvironment within the infected ganglionic tissues might help induce the expression of inhibitory ligands to dampen the activity of resident CD8^+^ T cells. An impaired activity of CD8^+^ T cells may help explain the frequent reactivation episodes. Some of the inhibitory receptor and their ligands that have been investigated in infected ganglions include PD1-PDL1, TIM-3-Galectin-9 (Frank et al., 2010; Reddy et al., 2011). IFN-γ presumably produced by resident T cells could help induce the ligands (Garcia-Diaz et al., 2017).

Stronger evidence for CD8^+^ T cells involvement in maintaining latency came from studies focusing on EBV latency (Dunne et al., 2002; Angelini et al., 2013). With EBV latency, some viral proteins are made which can provide the peptides for T cell recognition. Evidence indicates that the IFN-γ producing CD8^+^ T cells are critically involved for controlling γ-herpesviruses such as MHV68 (Steed et al., 2006). Infection of mice with MHV68 has provided useful insights into the contribution of viral reactive CD8^+^ T cells in the pathogenesis of γ-herpesviruses (Husain et al., 1999; Gredmark-Russ et al., 2008; Nash and Dutia, 2008; Freeman et al., 2010). Additional information on the role of CD8^+^ T cells in γ-herpesviruses' latency *in vivo* may come from the newly developed TCR transnuclear mouse model for MHV68 (Sehrawat et al., 2012).

## Immune system management by herpesviruses

Herpesviruses evade immune destruction using a number of strategies. These include infection of tissues with limited accessibility to immune mediators particularly for α-herpesviruses, establishment of latency that allows minimal immune recognition and numerous active immunomodulatory procedures intrinsic to herpesviruses. Many excellent reviews have discussed immune evasive or immune managemental properties of different herpesviruses (Ploegh, 1998; Tortorella et al., 2000; Orange et al., 2002; Hewitt, 2003; Rezaee et al., 2006). Table 1 summarizes many such properties. We briefly discuss how herpesviruses can manage the immune system to ensure their persistence.

**Table 1:** Immune management strategies used by herpesviruses.

**Virus**	**Derivative of virus involved**	**Evasion mechanism**	**Outcome**	**References**
Alpha herpes virus (HSV1)	Glycoprotein gI/gE heterodimer	Binds with Fc domain of IgG	Block complement activation and ADCC mediated cell killing	Lubinski et al., 1998, 2011
	Glycoprotein gC	Interacts with C3b and blocks C5 and P binding to C3b	Interfere with complement activation	Kapadia et al., 2002
	ICP47	Inhibits TAP mediated peptide transport	Impaired antigen processing and presentation	Hill et al., 1995; Tortorella et al., 2000
	Glycoprotein gJ and gD	Prevent apoptosis in infected epithelial cell	Viral can survive within infected cell	Zhou et al., 2000
	HSV-2 ICP10PK and UL14	Prevent apoptosis in infected epithelial and neuron cells		Perkins et al., 2002
	US3	Interacts with programmed cell death domain 4		Tortorella et al., 2000
	Y34.5 and US11	Inhibit the activity of antiviral Protein Kinase R (PKR)	Upregulation of viral protein translation	He et al., 1997; Poppers et al., 2000
	ICP0	Abrogates IRF3 mediated transcription regulation	Interfere host interferon signaling	Peng et al., 2009
	ICP27	Hamper nuclear accumulation of STAT-1	Interfere type-I IFN signaling	Peng et al., 2009
	US3	Blocks the IRF3 activation by hyperphosphorylating it	Inhibition of IFN-β production	Tortorella et al., 2000
	VP16	Inhibition of NF-κB activation		Peng et al., 2009
	UL36	Blocks IRF3 activation by deubiquitination of TRAF3		Peng et al., 2009
Beta herpes virus (HCMV)	UL18	Acts as a decoy for NK cell–MHCI homolog	Block NK cell mediated killing	
	US3, US10	Retention of MHCI in endoplasmic reticulum	Impaired antigen processing and presentation	Furman et al., 2002
	US2, US11	Degradation of MHCI and MHCII		Barel et al., 2003
	US6	Attacks the TAP complex and interfere with cytosolic peptide transport		Ahn et al., 1997; Hengel et al., 1997
	pp65	Inhibits proteasome activity		Odeberg et al., 2003
Gamma herpes virus (EBV)	BGLF5	Degradation of MHCI molecule mRNA	Impaired antigen processing and presentation	Rowe et al., 2007
	BILF1	Degrades the surface and on route MHCI molecules		Zuo et al., 2011b
	BNLF2	Blocks TAP-mediated peptide transport and MHCI retained in ER		Croft et al., 2009
	BZLF1	Downregulates the invariant chain expression for MHCII complex generation		Zuo et al., 2011b
	BZLF2	Creates stearic hindrance in MHCII and TCR interaction of CD4^+^ T cell		Ressing et al., 2003; Zuo et al., 2011b
	BDLF3	Proteasome pathway mediated downregulation of both MHC I and MHC II molecules		Zuo et al., 2011a
	EBNA-1	Interferes with proteasome activity during class I complex generation		Levitskaya et al., 1997; Münz et al., 2009
	vIL10	Downregulates the TAP activity and hampers in the class I molecule generation		Zeidler et al., 1997
	K5 protein of HHV-8	Downregulates the NK cell ligand ICAM-1 and B7.2	Block NK cell mediated killing	Ishido et al., 2000

*ICP47, infected cell protein 47; ICP, infected cell protein; ICP10PK, protein kinase activity of large subunit of HSV-2 ribonucleotide reductase protein; UL14, minor component of HSV-2 tegument; US2, US3, US6, US10, US11, VP16, pp65, UL14, UL18, and UL36, all are tegument protein domains; IRF3, IFN regulatory factor 3; US3, HSV specific serine/threonine protein kinase; TRAF3, TNF receptor associated factor 3; TAP, transporter associated with antigen processing; EBNA-1, Epstein-Barr nuclear antigen 1; vIL10, viral interleukin 10; ICAM-1, intercellular adhesion molecule 1; BGLF5, BILF1,BNLF2, BZLF1, BDLF3 are different proteins or open reading frames encoded by EBV and exhibit different activities*.

Herpesviruses can even establish a productive infection in the immune host. This fact reduces enthusiasm for vaccination strategies and most vaccine candidates have had only limited success. All herpesviruses blunt immunity either by interfering with immune induction or by producing anti-inflammatory molecules. For example, ICP47 of HSV and US6 of HCMV interact with the transporter of antigen presentation and processing (TAP) molecule thereby blocking efficient transportation of viral derived cytosolic peptides for loading on class I MHC (Hill et al., 1995; Ahn et al., 1997; Hengel et al., 1997). The HCMV protein pp65 can induce retention of class II MHC molecules in lysosomes for its destruction thereby minimizing its availability for surface display and limiting the T cell response (Ploegh, 1998; Odeberg et al., 2003). MCMV and HCMV can destabilize surface class I MHC by interacting with surface displayed β2 microglobulin leading to its down regulation (Jones and Sun, 1997; Halenius et al., 2015). A low level of MHC expression by infected cells could activate NK cells, but some herpesviruses have devised strategies to counteract NK cell mediated lysis of infected cells (Jonjić et al., 2008). These include modulation of host cell protein expression, encoding host homologs as well as viral proteins that could dampen NK cell responses (Grauwet et al., 2014; Campbell et al., 2015). The EBNA1 protein of EBV is not efficiently recognized by CD8^+^ T cells owing to its glycine-alanine repeat sequences which hinder the generation of immunogenic epitopes (Levitskaya et al., 1997; Münz and Moormann, 2008). Similarly, latency associated nuclear antigen (LANA) of KSHV and LANA-homologs of other γ-herpesviruses also interfere with antigen presentation (Coscoy, 2007). KSHV exhibit numerous immunomodulatory activities that range from interfering with components of the complement system, type I IFN signaling, in addition to impairing T and B cell responses as well as blocking apoptosis (Moore and Chang, 2003; Rezaee et al., 2006). Some herpesviruses encode for anti-inflammatory molecules (Spencer et al., 2002; Coscoy, 2007). For example, homologs of host receptors and anti-inflammatory molecules such as IL-10 and IL-35 are encoded by CMV and EBV, respectively (Birkenbach et al., 1993; Spencer et al., 2002; Rezaee et al., 2006; Rouse and Sehrawat, 2010).

Herpesviruses in general are excellent managers of the immune response and can successfully survive in the immune host. Since herpesviruses infect most individuals, an idea worth considering is how herpesviruses influence the outcome of other concurrent infections, cancers and grafts. Recent studies have shown that herpesviruses may, in fact, help fine-tune host immunity. This occurs by modulating one or more types of immune responses to make the host either more resistant or susceptible to other disease situations (Barton et al., 2007; White et al., 2010; Furman et al., 2015; Litjens et al., 2018) For example, some studies measured the influence of persisting β-herpesvirus (MCMV) or a γ-herpesvirus (MHV68) infection on the subsequent infection by *Listeria monocytogenes* and *Yersinia pestis*. Previously infected mice controlled these infections better than those not infected with MHV68 (Barton et al., 2007). The enhanced IFN-γ production, presumably by virus reactive T cells, activated macrophages, which then controlled bacterial growth. Enhanced NK cell responses as a result of MHV68 infection might also have participated in the control of these secondary infections (White et al., 2010). Prior infection with herpesviruses in humans as well as mice was also shown to promote immune response to a subsequent infection or the vaccination (Furman et al., 2015). CMV positive adults mounted a stronger anti-influenza virus CD8^+^ T cell response as compared to CMV negative individuals (Furman et al., 2015). Genital HSV2 infection promotes colonization of group B streptococcus locally in the genital tract (Cherpes et al., 2005). However, the molecular and cellular mechanisms involved have not been investigated but an anti-inflammatory milieu induced by the virus might have facilitated subsequent bacterial colonization. Another well established example of herpesvirus infection promoting secondary bacterial infection is VZV and invasive Group A streptococcus infections (Wilson et al., 1995; Laupland et al., 2000; Oyake et al., 2000). Severe lesions such as cellulitis, necrotizing fasciitis and sometimes endocarditis occur in people infected with VZV and Group A streptococcus infection (Zachariadou et al., 2014). A generalized immunosuppression caused by VZV infection seems to explain the outcome (Laskey et al., 2000). Cellular and molecular mechanisms for enhanced disease due to double infection are yet to be fully investigated.

Herpesviruses may also exhibit altered dynamics with their host during transplantation. Many humans receive transplantation to ameliorate malignant diseases or to restore organ functions. Iatrogenic immunosuppression is usually induced in patients undergoing solid organ transplants or hematopoietic stem cell transplants (HSCT). Such procedures often reactivate any persistent herpesvirus. Depending on the herpesvirus involved, the outcome of transplantation procedures might vary. For example, HSCT aimed at improving the prognosis of acute myeloid leukemia (AML), induced reactivation of CMV which then expanded a subset of donor derived NKG2C expressing NK cells as well as γδ-T cells of a particular phenotype (Vδ2^−ve^ γδ-T cells) (Litjens et al., 2018). These cells helped control the relapse of leukemic episodes thereby benefiting the host. CMV in transplant patients can also cause harmful effects such as genital tract disease, hepatitis, encephalitis or retinitis. In some patients undergoing HSCT to improve the outcome of Hodgkin lymphoma, HHV-6 gets reactivated (Drobyski et al., 1994). The reactivated virus eventually resulted in meningioencephalitis and death. Many a time transplant tissues could also serve as the source of transmitting herpesviruses in the recipients (Openshaw et al., 1995; Remeijer et al., 2001). Therefore, in patients undergoing transplantation, anti-herpesviral drugs are usually infused (McIntosh et al., 2016).

## Disruptors of host–herpesvirus–harmony

The large majority of people harbor herpesviruses as part of their virome (Virgin et al., 2009) and these infections usually do not cause major harm in healthy adult host. The scenario can change, however, under several circumstances such as when the host has defects in innate or adaptive immunity. Such problems normally become evident in neonates, children and elderly; transplant patients as well as cancer patients or patients having one or more concurrent infections. Such issues are discussed subsequently.

### Genetic insufficiencies can result in severe herpetic disease due to primary infections

A compromised or dysfunctional immune system invariably results in severe disease outcome due to herpesvirus infections. Individuals with defects of innate immunity usually fail to control most herpesviruses (Fitzgerald et al., 1985; Krug et al., 2004). The activity of NK cells and more importantly signaling through type I interferons (IFNs) are critical in providing anti-herpesvirus defense (Fitzgerald et al., 1985; Smith H. R. C. et al., 2002; Krug et al., 2004; Takeuchi and Akira, 2009). Most studies have come from animal models, but evidence from human studies also suggest for the role of innate immune mediators in defense against herpesvirus infections (Dupuis et al., 2003; Jost and Altfeld, 2013). Genetic deficiency or mutations in TLR3 and UNC93B can result in herpes simplex virus encephalitis and mortality due to impaired type I IFN response (Casrouge et al., 2006; Zhang et al., 2007; Iwasaki, 2012; Rosato et al., 2015). One of the typical clinical presentations of HSV 1 infection is an occurrence of herpes simplex labialis (HLS). However, only 20-30% of infected individuals exhibit HLS, therefore attempts were made to identify genetic susceptibility loci (Kriesel et al., 2011). Specifically, two single nucleotide polymorphism (SNP) within the chromosome 21orf 91 (C21orf91) exhibited a strong association with disease development. This reading frame, also called as cold sore susceptibility gene (CSSG1), encoded for a cytosolic expressing protein whose function still remains unknown. The transcripts for this gene were also recorded in other cells such as immune cells and ganglionic cells (Kriesel et al., 2011). Therefore, there is possibility that the protein encoded might have critical function in deciding the outcome of herpesvirus-mediated encephalitis which also exhibit some associations with genetic immunodeficiencies. Additional genetic mutations responsible for defective innate immunity against herpesviruses include mutations of the NF-kB essential modulator gene as well as mutation in GATA2 (Zandi et al., 1997; Dropulic and Cohen, 2011). An extended list of genes responsible for severe herpesvirus infections is provided in Table 2. Many of these deficiencies result in uncontrolled and aberrant immune responses in infected individuals leading to immunopathologies (Parvaneh et al., 2013). Some of these conditions can also cause neoplastic transformations, especially in γ-herpesvirus infected individuals (Parvaneh et al., 2013; Ruffner et al., 2017). Defects in some anti-inflammatory response such as the inability of host to mount an appropriate anti-inflammatory response can also result in severe immunopathologies caused by some herpesviruses. Extensively studied anti-inflammatory mechanisms include the cytokine IL-10 and regulatory T cells expressing transcription factor Foxp3 (Lund et al., 2008; Sarangi et al., 2008; Rouse and Sehrawat, 2010; Veiga-Parga et al., 2013; Sehrawat and Rouse, 2017).

**Table 2:** Primary immunodeficiencies and the outcome of herpesviral infections.

**Host molecules**	**Virus involved**	**Outcome**	**References**
PRF1, STXBP2	EBV	Inefficient immune response and leads to the familial HLHs	Reviewed in Parvaneh et al. (2013)
ITK	EBV	B-cell lymphoproliferation, Hodgkin's lymphoma, hepatosplenomegaly, cytopenia, hypogammaglobulinaemia, and high viral load in host	Huck et al., 2009; Stepensky et al., 2011; Linka et al., 2012
CD27	EBV	Hypogammaglobulinaemia, reduced memory B cell count, diminished CD8^+^ T cell and iNKT cell function	van Montfrans et al., 2012; Salzer et al., 2013
MAGT1	EBV	Abrogates T cell activation and reduced CD4^+^ T cell count	Li et al., 2011
STK4	EBV	Hypergammaglobulinaemia, reduced TCR repertoire, and naive T cell count, enhanced recurrent infection	Abdollahpour et al., 2012; Nehme et al., 2012
CORO1A	EBV	B-cell lymphoproliferation	Moshous et al., 2013
SH2D1A	EBV	HLH	Marsh and Filipovich, 2011
MCM4	EBV	EBV lymphoma	Reviewed in Ruffner et al. (2017)
OX40	KSHV	Enhanced susceptibility to mycobacteria	
IFNGR1	KSHV, VZV	Inadequate cytokine signaling	
DOCK8, NEMO, GATA2, STAT1 GOF, STK4, WHIM	HSV, VZV	Chronic infection, elevated cutaneous coinfection	
STAT3 LOF	VZV	Altered T cell population leading to immunopathological response	
CXCR4	HSV	Abnormal neutrophils, pancytopenia	
WAS	HSV	Eczema, thrombocytopenia	
UNC-93B	HSV	Impaired type I and type II IFN responses, HSE	Casrouge et al., 2006
TLR3	HSV	Impaired type I and type II IFN responses, HSE	Casrouge et al., 2006
PRF1	CMV	Diminished CD8^+^ T cell and NK cell mediated killing	Kägi et al., 1996
UNC13D	CMV	Familial HLH	Crozat et al., 2007

*PRF1, Perforin 1; STXBP2, Syntaxin binding protein 2; ITK, interleukin-2 inducible T cell kinase; EBV, Epstein-Barr virus; MAGT1, magnesium transporter 1; STK4, serine/threonine protein kinase 4; CORO1A, Coronin-1A; SH2D1A, Src homology 2 domain-containing gene 1 A; HLH, hemophagocytic lymphohistiocytosis; XIAP, X-linked inhibitor of apoptosis protein; MCM4, minichromosome maintenance protein 4; iNKT, invariant natural killer T cell; KSHV, kaposi sarcoma herpes virus; IFNGR1, interferon gamma receptor 1; DOCK8, dedicator of cytokinesis 8; NEMO, NF-κ B essential modulator; STAT1, signal transducer and activator of transcription 1; GOF, gain of function; LOF, loss of function; STK4, serine/threonine protein kinase 4; WHIM, warts, hypogammaglobulinemia, infections, and myelokathexis; VZV, varicella zoster virus, CMV, cytomegalovirus, CXCR4, C-X-C chemokine receptor 4; WAS, Wiskott-Aldrich syndrome; HSE, herpes simplex encephalitis; TLR3, toll like receptor 3*.

All primary immunodeficiencies invariably result in severe disease resulting from primary herpesvirus infections before the onset of a harmonious relationship with virus.

### Interaction of herpesviruses with HIV can alter the outcome of both infections

The emergence of human immunodeficiency virus - acquired immunodeficiency syndrome (HIV-AIDS) 40 years ago seems to have altered the relationship of several herpesviruses with their host. Indeed, co-infection involving HIV and herpesviruses are frequent. Kaposi sarcomas on the skin caused by HHV-8 or KSHV readily became evident in HIV infected individuals in the pre-treatment era (Sepkowitz, 2001). Another common problem in the pre-treatment era was retinitis caused by HCMV infection, a lesion almost never seen in treated AIDS patients (Salzberger et al., 2005). Enhanced expression of proinflammatory molecules such as IL-1β, TNF-α, CCR5, vCXCL1, vCXCL2 in concurrently infected individuals facilitated the recruitment of more inflammatory cells that precipitated HCMV retinitis (Safdar et al., 2002; Heiden et al., 2007; Lichtner et al., 2015). Similarly individuals receiving profound iatrogenic immunosuppressive therapies to achieve acceptance of graft tissues also tend to develop severe HCMV infections (Dowling et al., 1976). Zostriform infection (commonly known as shingles) caused by the reactivation of VZV infection is usually a problem of aged individuals but untreated younger AIDS patients also exhibit severe manifestation (Buchbinder et al., 1992; Leppard and Naburi, 1998). There are instances where co-infection of herpesvirus and HIV can aggravate the outcome of HIV infections (Kucera et al., 1990; Regezi et al., 1996; Tobian and Quinn, 2009). Multiple explanations have been proposed. For example, genital ulcers caused by herpesviruses (particularly HSV 2) may disrupt the integrity of the mucosa which can facilitate HIV infection of infiltrating T cells and macrophages (Kucera et al., 1990; Tobian and Quinn, 2009). Although HIV can infect resting immune cells, activated CD4^+^ T cells are more prone to infection (Okoye and Picker, 2013). Episodic reactivation of HSV expands activated CD4^+^ T cell population which can be easily infected by HIV (Okoye and Picker, 2013). With the loss of T cells, host's susceptibility to HSV and other opportunistic infections increases (Bartlett et al., 2012; Okoye and Picker, 2013).

Another idea proposed for enhanced susceptibility of HIV by prior herpesvirus infections included an accelerated phagocytosis and internalization of HIV (Takeda et al., 1988; McKeating et al., 1990). Thus, HSV 1 or CMV infections were shown to enhance Fc receptor expression on some cells (Westmoreland and Watkins, 1974; McKeating et al., 1990). Elevated FcR expression can efficiently bind to the Fc portion of an antibody molecule some of which can also form complex with HIV. The immune complexes thus formed were internalized more efficiently. However, this could only be a factor when the pre-existing anti-HIV antibodies unable to neutralize the virus are present in the host. In addition this phenomenon may be more relevant to amplify the infection. Some suggest that the direct interaction of components of herpesviruses with HIV can enhance HIV replication in infected cells (Ostrove et al., 1987; Gimble et al., 1988; Albrecht et al., 1989; Margolis et al., 1992). For instance, ICP0 and ICP4 of HSV can interact with the long terminal repeats (LTRs) of HIV to enhance its replication (Gimble et al., 1988; Margolis et al., 1992). Another theory proposes that concurrent herpesviruses and HIV infections result in the selection of new HIV variants with greater infectivity toward new cell types (Grivel et al., 2001). For example, HIV variants (X4) that predominantly infect CD4^+^ T cells emerge more frequently in patients co-infected with HHV-6 and HHV-7 than in those infected with HIV alone (Grivel et al., 2001). Although detailed molecular mechanisms to explain such observations are lacking but some studies suggested the involvement of tissue resident inflammatory cells that included CD4^+^ T cells and dendritic cells expressing receptor for HIV such as DC-SIGN or CD123 in previously HSV 2 infected individuals (Zhu et al., 2009). Intriguingly such cells persisted at tissue sites even after virus is controlled effectively by anti-viral treatment (Zhu et al., 2009). The prolonged persistence of immune cells in healed genital ulcer could therefore also help facilitate HIV infection. An association between EBV induced Burkitt's lymphoma (BL) and HIV is reasonably well established (Beral, 1991). BL is a rare cancer of children and is endemic in some African countries. HIV induces hyper proliferation of B cells which then can be infected by EBV (Grogg et al., 2007). This cellular mechanism could enhance lymphoma development.

We can conclude that individuals having co-infection with one or more herpesviruses and HIV exhibit more severe disease by either agent. Accordingly, the clinical management of such cases would require treating both infections simultaneously.

### Is there a link between herpesvirus infections and malaria?

The severe consequence of co-infection involving the γ-herpesvirus (EBV) and malaria is well established (Epstein et al., 1964). The interaction could promote the development of Burkitt's lymphoma (BL). Antigens derived from *Plasmodium falciparum (Pf)*, the causative agent of a severe form of malaria, induce polyclonal B cell activation (Chêne et al., 2007). B cells thus activated are more permissive to EBV infection and undergo hyper proliferation. *Pf* infection is also known to down regulate the expression of activation induced cytidine deaminase, an enzyme responsible for c-myc translocation in latently EBV infected B cells (Torgbor et al., 2014). This step is considered critical for the development of cancer. A possible role of upregulated TLR9 by EBV latently infected B cells and its subsequent activation by *Pf* derived agonists is also proposed as a mechanism for hyperproliferation of B cells (Crompton et al., 2009; Iskra et al., 2010). Co-infection of EBV and *Pf*, especially in children, creates an anti-inflammatory environment with enhanced IL-10 production. This could blunt the activity of CD8^+^ T cells which would fail to clear infected B cells (Peyron et al., 1994; Medina et al., 2011). In addition, EBV EBNA1 protein is not a potent stimulator of protective CD8^+^ T cells and this effect is further enhanced by co-infection with *Pf* (Levitskaya et al., 1997). Some studies were performed in mice to understand the cellular and molecular mechanism of enhanced severity of coinfection involving γ-herpesvirus and *Plasmodium*. Mice coinfected with MHV68 and *Plasmodium* displayed severe disease (Matar et al., 2015). An acute but not latent infection of mice with γ-herpesvirus failed to control the subsequent non-lethal *P. yoelii* infection (Haque et al., 2004; Matar et al., 2015). A compromised anti-plasmodial humoral immune response mediated by the M2 protein of MHV68 was suggested to explain the phenomenon (Matar et al., 2015). Other factors such as the altered responses of immune cells other than those of the adaptive arm were not investigated and could be also involved.

### Heterologous immunity and herpesvirus infections

An accumulating body of evidence shows that the outcome of a herpesvirus infection can be influenced by the host's past experience with other infectious agents, as well as the microflora, which colonize the gut, skin and other sites (Robinson and Pfeiffer, 2014; Shannon et al., 2017). Cross-reactive B and or T cell responses to unrelated pathogens can be responsible for the altered outcome of a subsequent infection. This is known as heterologous immunity. A series of elegant studies in mice with multiple infecting viruses was done by Welsh and Selin group (Welsh and Selin, 2002; Clute et al., 2005; Welsh and Fujinami, 2007; Welsh et al., 2010). Their studies provided some rules to explain why a second infection has variable outcome in terms of disease expression. Accordingly, one of the influenza virus epitope (M1_58−66_) and EBV derived epitopes (BMLF1_280−288_ and BRLF1_109−117_) were shown to be cross-reactive. The severity of mononucleosis correlated with specific expansion of BMLF1_280−288_ specific CD8^+^ T cells (Clute et al., 2005). The presence of a particular MHC haplotype (HLA-A2^+^) was suggested to account for the skewed response and lymphocytosis. The possibility of clinical mononucleosis (kissing disease) occurring after primary infection with EBV is more common in young adults who have been previously infected with influenza virus and generated a particular type of antibody response (McClain et al., 2003). Additional evidence where herpesvirus infections could influence responses to heterologous infections as well as to other diseases such as allergies, metabolic diseases and perhaps some cancers are awaited.

### Harmony changes to cacophony when herpesviruses infect a non-native host

During the course of evolution, most herpesviruses have adapted to a single or a limited number of host species. Nevertheless, when for a variety of reasons they happen to infect non-native species, aberrant and severe disease can result. A common example is HSV in mice, a popular model to study HSV pathogenesis. In mice, HSV does not behave as it does in its natural human host. Many strains are highly virulent in mice and cause lethal encephalitis particularly when higher doses are used for infection. Encephalitis is a very rare outcome in healthy humans and not influenced by viral strain type. When some herpesviruses infect a non-native species, encephalitis can result. For example humans accidentally infected with herpes B virus, an α-herpesvirus of monkeys, can suffer from lethal encephalitis (Wozniakowski and Samorek-Salamonowicz, 2015). Similarly pseudorabies virus of pigs causes encephalitis in cows (Crandell et al., 1982). Another example include a β-herpesvirus, elephant endotheliotropic herpesvirus (EEHV), which normally infects African elephants but is usually innocuous in the adult animals (Richman et al., 1999; Fickel et al., 2001). When the virus happens to infect young animals or Asian elephants, the disease is invariably severe (Richman et al., 2000; Fickel et al., 2001). Why encephalitis occurs so commonly in non-native species as compared to the native species is not known. The virus may be arrested in ganglionic neurons in latent form and fail to spread anterograde to the CNS. This retention may not happen in a non-native host for reasons still not clear.

### Do herpesviruses influence pathogenesis of autoimmune diseases?

Multiple events contribute to the onset, progression and severity of autoimmune diseases. Many have advocated that herpesviruses can be associated with several autoimmune diseases, perhaps acting as triggering agents for their onset (Münz et al., 2009; Getts et al., 2013). However, few if any investigators subscribe to the hypothesis that some human autoimmunities are directly caused by herpesviruses or any other virus infection. Circumstantial evidence suggest that several herpesviruses, particularly EBV and HHV-6 could initiate the onset of multiple sclerosis, but such ideas have never been confirmed independently (Wucherpfennig and Strominger, 1995; Poole et al., 2006; Lünemann et al., 2007, 2008; Münz et al., 2009). It was advocated that the HSV induced ocular lesion, stromal keratitis, represented an autoimmune lesion during its chronic phase (Zhao et al., 1998). Evidence to support this hypothesis was presented using a mouse model in which molecular mimicry between a protein of HSV (UL-6) and an auto-antigen expressed in the cornea was suggested to cause the disease (Zhao et al., 1998). Such ideas were never independently confirmed and some data strongly argued against this hypothesis (Deshpande et al., 2001). At this stage of investigation, it is probably safe to assume that while there is no compelling evidence that herpesviruses can directly cause one or more autoimmune diseases, the viruses might serve as cofactors in the pathogenesis of autoimmune diseases.

## Conclusions

For most of us with a normally functioning immune system, exposure to and living with many herpesviruses has no major consequences. We usually develop a harmonious relationship with multiple herpesviruses that persistently infect us. Problems arise mainly with immune immaturity or when it declines with age or is dysregulated. The latter occurs most commonly as a consequence of cancer, infections with some pathogens, or immunosuppressive therapy to control transplants and tissue damaging lesions. A particular problem that changed the face of herpesvirus infections was the emergence of HIV, especially in the era before effective antiviral therapy. Fortunately, we now have better mechanistic understanding of the circumstances, which disrupt herpesvirus-host-harmony and may well be poised to exploit such information for better management of such situations. It has also become evident that herpesviruses form part of our virome and this can impact on susceptibility to other infections and disease producing agencies. Whether or not the composition of our virome can help explain the variability of the outcome of many herpetic diseases such as development of zosteriform infection, post-herpetic neuralgia as well as the severity of eye disease remains investigated.

A contentious issue in the herpesvirus field is the development of vaccines against member viruses. Indeed, studies in mice with almost any form of HSV vaccine protect them from disease, yet no vaccine against HSV in humans induces effective immunity, at least when subjected to double bind independent evaluation (Koelle and Corey, 2003). There are many enthusiastic advocates for universal vaccines against herpesviruses but our own view is that the prophylactic vaccines against some herpesviruses could be useful only under special circumstances. These include transplant patients treated with immunosuppressant drugs and defects in one or more aspects of immunity. What might really be useful against some herpesviruses is a therapeutic vaccine that could rewrite the language of immune responsiveness. This is because herpesviruses are so ubiquitous and their unmatched prevalence in general population. Making an inflammatory tissue damaging response into one that is far more benign particularly against pathogenic epitopes represents an appealing idea. However, this would require the identification of those antigens, which are predominantly pathogenic rather than protective. Progress in this area has been less than impressive, but we expect useful discovery in this field.

## Author contributions

SS has collected data, surveyed literature, written, and edited the MS. DK has surveyed literature, collected information and written the MS. BR help logically present ideas, written, and edited the MS.

### Conflict of interest statement

The authors declare that the research was conducted in the absence of any commercial or financial relationships that could be construed as a potential conflict of interest.
